# Generation Z involvement, cultural identity, and purchase intention in the homestay context: Evidence from tea-culture homestays

**DOI:** 10.1371/journal.pone.0348785

**Published:** 2026-05-06

**Authors:** Qing Zhang, Huazhen Sun, Qiuyan Lin, Weifeng Guo

**Affiliations:** School of Tourism, Wuyi University, Nanping, China; Telkom University, INDONESIA

## Abstract

This study examines how Generation Z involvement influences purchase intention for tea-culture homestays and whether cultural identity mediates this relationship. The attention of generation Z consumers to the cultural connotation of homestay is increasing, which has an impact on the subsequent development and continuous operation of homestay. This paper applies the theory of involvement and cultural identity, takes generation Z consumers as the research group, explores the influence of generation Z consumers’ involvement and cultural identity on their purchase intention, and studies the mediating role of cultural identity in this process, thus constructs a model of the relationship between generation Z consumers’ involvement, cultural identity and the influence of purchase intention of tea culture homestay, and applies SPSS24.0 and AMOS24.0 to analyze the data for empirical verification. data analysis for empirical verification. It is found that: (1) the degree of involvement of the generation Z of consumers has a significant positive effect on the cultural identity and purchase intention of tea culture homestay; (2) the cultural identity of the generation Z of consumers on tea culture homestay can positively affect the purchase intention of the consumers; (3) there is a partially mediating effect of the cultural identity in the degree of involvement of the generation Z of consumers and the purchase intention of the consumers. Finally, this paper puts forward relevant suggestions for the research conclusions.

## 1. Introduction

In recent years, with the rapid economic growth and rising income levels, consumer preferences in China have undergone a significant transformation—from satisfying basic material needs to pursuing richer spiritual and cultural experiences. homestay accommodations have emerged as a popular choice among consumers seeking immersive and personalized stays [1,2]. Unlike traditional hotels, homestay often incorporate local cultural elements through architectural style, interior decoration, and owner-curated experiences, thereby serving as tangible representations of regional identity and lifestyle aesthetics [3].

Generation Z gradually enters the consumer market and becomes the core force of the consumer market, and occupies a certain strength for the consumption of homestay. Influenced by the times, generation Z’s requirements for consumption are also rising, and they pay more attention to the consumption experience while pursuing personalization [4,5]. They are no longer satisfied with simply pursuing material satisfaction, but pay more attention to spiritual enjoyment, which is often a derivative of service value. In the eyes of this generation Z, hobbies and interests are one of the most important factors in their input points. Through the love and affection for things, to pay for their favorite things [6,7]. For generation Z, a certain cultural connotation will be more attractive to them for consumption [8,9]. How to stimulate generation Z of consumers to identify with the culture of the homestay, and then enhance the consumer’s willingness to buy, is the core problem of this research.

Involvement Theory can be used as a variable in psychological state, which refers to the degree of an individual’s inner drive or concern [10,11]. When an individual is exposed to some special situations or stimuli, the inner drive or concern will be triggered, making the individual feel how much a certain thing is related to him or her. Consumer involvement as an internal activity of consumers, which is continuous and ever-changing [12]. The degree of involvement has an important influence on purchasing decisions, which is mainly based on interests, goals and needs [13]. The higher the level of tourism consumer participation, the higher the perceived value of cultural and creative products [14,15]. Tourism involvement has a greater impact on the emotional value of tourism cultural and creative products, while product involvement has a greater impact on their functional value. However, there are few studies on the use of involvement in the choice of homestay in generation Z.

Cultural identity is a social psychological process that involves an individual’s sense of belonging and inner commitment to the culture and cultural group to which he or she belongs, in order to acquire, maintain and innovate his or her own cultural attributes [16]. Some scholars conducted empirical research on tourist involvement in different situations and found that cultural identity is an important factor affecting tourist involvement [17–19]. Other scholars explored cultural identity in the tourism environment, from the perspectives of tourists’ cultural identity with the destination and local residents’ cultural identity with their own ethnic group [20].Few studies have analyzed the role of cultural identity in homestay purchases.

To sum up, by introducing the theory of involvement and the theory of cultural identity, this paper constructs a model of the influence of involvement, cultural identity and consumer purchase intention of generation Z consumers by taking the cultural identity of generation Z consumers to homestay as the key mediating factor affecting their purchase intention, and analyzes the effect of its role, in order to explore the relationship between the identification of generation Z consumers to the culture of homestay and the purchase intention of consumers, with a view to providing some lessons for the sustainable management of homestay operation and management. In order to provide some lessons for the sustainable development of homestay management.

## 2. Materials and methods

### 2.1. Research hypotheses

(1) Generation Z’s Consumer Involvement and Consumer Purchase Intention

Consumers’ involvement with content has an impact on their motivation to buy, and as their involvement with the product deepens over time, they gradually develop perceptions and memories of the product, which can lead to a desire to purchase it [11,21,22]. Many consumers are influenced by the characteristics of the product itself and the level of knowledge about the product before purchase, thus showing different levels of involvement [23,24]. Peoples’ willingness to purchase tourism cultural and creative products is positively influenced by the degree of involvement, and both the degree of tourism involvement and the degree of product involvement can have a positive predictive effect on it [11]. Fast food, as a retrospective system, its purchase intention is directly proportional to the consumer’s degree of involvement, that is to say, the higher the degree of involvement, the greater the uncertainty felt by the consumer, but the stronger its purchase intention [25]. Liu Jianxin (2010) and others pointed out that consumer involvement is caused by the situation or stimulus, which refers to the degree of consumer awareness of a consumer object according to personal needs, interests, values or external stimuli to perceive the relevance of the consumer object and itself, which can be analyzed from the intensity, direction and persistence of the three aspects in order to affect the information search and willingness to buy [26]. Others studied from the perspective of perceived value, and concluded that consumer purchase intention is closely related to product involvement and perceived value, and purchase intention increases with increased involvement [27]. On this basis, relevant hypotheses are proposed. When the enterprise establishes a strong degree of recognition in the minds of consumers, their desire to buy will also be enhanced [28]. In research, involvement degree is used as a predictor variable to study the consumer’s purchase decision process, which is a very common phenomenon to a large extent. Generally speaking, involvement degree indicates that there is a personal correlation between the product and the consumer’s needs, and that the consumer is willing to invest more time in product information search, which results in a certain willingness to purchase [29–32]. Accordingly, the following hypotheses are proposed in this study:

Hypothesis 1 (H1). Generation Z’s involvement positively influences purchase intention

(2) Generation Z’s Consumer Involvement and Cultural Identity

As consumers’ involvement increases, their identification with the cultural domains they are involved in increases. In a study conducted by Wu Xiaoxu (2010), it was found that there is a significant correlation between tourist involvement and place attachment among rural tourists, and that the more involvement there is, the stronger the attachment to the destination [33,34].Hwang found a significant positive correlation between tourists’ involvement behaviors and place attachment [33,35]. The degree of tourists’ involvement in Xuzhou Han cultural tourism area has a significant impact on the emotional attachment to the place [36]. Tourist involvement significantly and positively affects cultural identity, destination image and revisit intention [37]. According to Cui Xinjian (2004), the core of identity lies in cultural identity, which coexists in various forms of identification, and culture is a symbol of a specific identity and role, so there is a cultural element embedded in place attachment. That is to say, involvement is likely to have an impact on cultural identity, and a number of scholars have already conducted empirical research on the relationship between the two [38]. The study of Multiple studies have argued that more involvement and a higher degree of involvement result in a higher level of identification with the culture involved [39–41]. Based on this, this paper proposes the following hypotheses:

Hypothesis 2 (H2). Generation Z’s involvement positively influences cultural identity

(3) Cultural Identity and Consumer Purchase Intention

A large number of previous studies have shown that local cultural identity can make consumers have a better willingness to buy local goods and so on. Su Yong and Li Zhina (2008) found that consumers’ foreign cultural identity can directly enhance their desire to buy products from their home country, while consumers’ foreign cultural identity can also directly enhance their evaluation of foreign products, and indirectly enhance their willingness to buy foreign products through their evaluation of foreign products [42,43]. While a consumer has a higher degree of identification with a country’s culture, they are more inclined to buy products from that country [44]. A study showed that regional cultural identity plays a positive role in consumers’ purchase intention [45]. The cultural identity of traditional Chinese medicine has a positive impact on consumers’ decision-making to purchase traditional Chinese medicine cultural and creative products; the purchase motivation of traditional Chinese medicine cultural and creative products is significantly and positively influenced by the value of use and cost-effectiveness [46]. According to the results of several survey respondents, consumers’ willingness to purchase the product is positively correlated with their cultural identity, i.e., the higher the cultural identity, the stronger the willingness to purchase [47–49]. On this basis, this paper puts forward the following hypotheses:

Hypothesis 3 (H3). Generation Z’s cultural identity positively influences purchase intention

(4) Mediating variables of cultural identity

In studying the relationship between consumer involvement and purchase intention, some scholars believe that consumer involvement directly affects consumer purchase intention, but some scholars believe that consumer involvement can be achieved through perceived value, word-of-mouth, and brand relationship quality [50–52]and other ways to have an indirect effect on consumer purchase intention [53,54]. According to the path of “cognition-attitude-behavior”, as generation Z of consumers continue to invest in homestay, they will gradually form their identity and belonging to the homestay culture. On the basis of this recognition, they will pay more attention to the experience quality and service quality of the homestay service, and will actively participate in the management of the homestay in order to improve their own satisfaction. The subsequent behavior of generation Z consumers is continuously influenced by the sense of identity, which includes the positive evaluation of the accommodation experience, as well as the transmission of their own feelings to others, so as to obtain the recognition of others, which indirectly affects their willingness to buy [27,50]. Based on this, this paper proposes the following hypotheses:

Hypothesis 4 (H4). Generation Z’s cultural identity mediates the relationship between involvement and purchase intention

**Fig 1:**
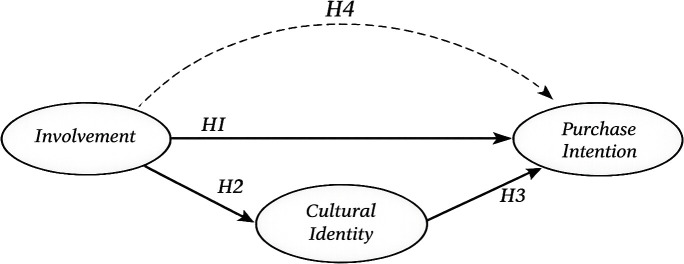
Conceptual model.

The proposed conceptual research model is shown in Fig 1.

## 3. Data source and method

### 3.1. Study sites

Wuyishan City, a county-level city under the jurisdiction of Fujian Province, is administered by Nanping City. It is located in the northwest of Fujian Province and belongs to the mid-subtropical monsoon humid climate zone. The total area is 2,813 square kilometers. Wuyishan City has unique cultural resources such as Pengzu culture, Minyue culture, Zhuzi culture, tea culture, and Liuyong culture. In the Song Dynasty, it was called “Minbang Zou Lu” and “Daonan Li Ku” by Confucianists. It is the birthplace of Zhuzi’s Neo-Confucianism. It is the birthplace of oolong tea and black tea, two of the world’s six major teas. It is the starting point of the “Tea Horse Road” and the first “Hometown of Chinese Tea Culture and Art”. The production skills of Wuyi Rock Tea (Dahongpao) have been listed as the first batch of national intangible cultural heritage.

### 3.2. Survey design

To confirm content validity, a team of three management professors—two Chinese and one English—converted the English scales into Chinese counterparts and then translated them back into English using the back-translation process.This paper mainly uses questionnaires to obtain survey data, and the variables mentioned in the paper are all based on a five-level Likert scale, in which the first to the fifth levels indicate strongly disagree to strongly agree, respectively. The design of the scales all refer to the mature scales that have been empirically tested at home and abroad, and combined with today’s context, the scales involved in each variable are situationally adapted to ensure the reliability and validity of the questionnaire content. The questionnaire can efficiently obtain first-hand data, so it can more accurately carry out the next step of research on the variables, and the whole questionnaire is roughly divided into two modules:

The first module, which focuses on the collection of basic information data, including gender, age, education, monthly income, occupation, number of times staying in tea culture lodges, and ways of learning about tea culture lodges.

The second module, which contains three variables to be studied, has a total of five items on generation Z involvement and seven items on cultural identity.

### 3.3. Measurement scales

Measurement of generation Z’s consumer involvement variables, this paper utilizes Li Qianghong et al. [50].The five measurement indexes studied, dividing involvement into cognitive involvement and affective involvement, can be used to obtain a measurement scale for the level of consumer involvement.

Measurement of cultural identity variables, the current study builds on previous research by defining cultural identity as a role relationship between individuals within the same characteristic group, and in particular, in this study it is the collective identity established by generation Z of consumers with respect to the salient features of the tea culture homestay. Using Luhtanen Crocker (1992) [55]to measure cultural identity, the following measurement scale on cultural identity can be obtained by using affective and evaluative as the analyzed dimensions of cultural identity.

Measurement of consumer purchase intention variables, purchase intention reference Hwang & Zhang (2018) [56]The metric used in the study mainly measures consumers’ willingness to stay in homestay through 4 question items into.

### 3.3. Data collection

In order to measure the scale reliability level and validity level of the questionnaire of this study, a small pre-survey was conducted before the formal survey, and the distribution period was from December 20, 2022 to January 10, 2023. Since there are three main variables in this paper, totaling 16 measurement questions, the sample size of the pre-survey in this study should be at least 32. On this basis, 102 questionnaires were distributed in the pre-survey stage, 100 were returned with a 98% recovery rate, and 98 questionnaires were valid with an effective rate of 96.07%. Based on this, this study utilizes SPSS24.0 statistical software to conduct a series of reliability and validity tests for the above 98 questionnaires. The Cronbach’s Alpha reliability coefficient alpha of the measurement items of the variables in this study are higher than 0.7, which indicates that the measurement items of the variables have consistency and good reliability. At the same time, the KMO values of each variable are greater than 0.7, which indicates that the variables in this study have a good level of validity.

In this paper, the main target of the questionnaire is generation Z of consumers, and the period of distribution is from January 20, 2023 to March 15, 2023 to collect the data of the questionnaire. The respondents were initially informed of the research purpose and the survey’s anonymous and voluntary nature. The questionnaire was only delivered to the participant once they orally consented to complete it and attested to being older than eighteen. Afterwards, the recovered questionnaires were statistically organized and analyzed for research. Several social platforms such as WeChat group chat, QQ group chat, and Xiaohongshu were utilized for data collection.

A total of 310 questionnaires were distributed and 308 questionnaires were recovered, with a recovery rate as high as 99.3%. After rigorous screening, questionnaires that obviously did not meet the criteria were excluded, and finally 300 valid questionnaires were obtained, with an effective rate as high as 97.4%.

## 4. Results

### 4.1. Descriptive statistical analysis of the sample

Descriptive statistical analysis focuses on describing the data of the questionnaire respondents. As far as the gender distribution is concerned, the number of males is 144 and the number of females is 156, the proportion of males is 48% and the proportion of females is 52%, which is roughly equal to the proportion of males and females. The recovered questionnaires were statistically organized and analyzed for research. The survey respondents were mainly concentrated between the ages of 21 and 29, while the proportions of those between18–20 and between the ages of 30 and 39 were 23% and 14%, respectively, mainly concentrated in generation Z. The education level of the survey respondents is generally high, with 58.3% of the respondents having a bachelor’s degree, 3% having less than junior high school, 13.7% having senior high school or middle school, 19% having junior college, and 6% having postgraduate and upper-level education, which suggests that the education level of generation Z is already more than half of the bachelor’s level. In terms of occupation type, school students had the highest proportion of respondents, followed by corporate employees and other industries, while professional and technical personnel had the smallest proportion. According to the statistics of monthly disposable income, 36% of the respondents had a monthly income of less than 2,000 yuan, 19.3% had a monthly income of 2001–4,000 yuan, 22% had a monthly income of 4,001–6,000 yuan, 10% had a monthly income of 6,001–8,000 yuan, 6.7% had a monthly income of 8,001–10,000 yuan, and 6% had a monthly income of more than 10,000 yuan.

### 4.2. Reliability and validity tests

The model was fitted using AMOS 24.0, and the fit indices were CMIN/DF = 2.212, NFI = 0.929, CFI = 0.960, TLI = 0.952, and RMSEA = 0.064(Table 1), with each of the fit indices having a significant fit and a high degree of fit. The standardized factor loadings of generation Z involvement, cultural identity and consumer purchase intention are between 0.665 and 0.856, respectively, which are all greater than the standard threshold of 0.5, which indicates that these observed variables have strong explanatory power for the latent variables. The Cronbach’s α coefficient for the overall measurement scale was determined to be 0.954. Additionally, the Cronbach’s α values for the individual measurement scales ranged from 0.786 to 0.969. These values surpass the recommended threshold of 0.7, indicating that the measurement scale exhibits strong reliability and internal consistency [57].

**Table 1:** Model fit indices.

	CMIN/DF	GFI	NFI	TLI	CFI	RMSEA
measured value	2.212	0.909	0.929	0.952	0.96	0.064
adaptation value	<3	>=0.9	>=0.9	>=0.9	>=0.9	<0.08

The loading values for each latent variable range from 0.665 to 0.856, all of which surpass the standard threshold of 0.60. Additionally, the composite reliability (CR) values fall between 0.563 and 0.724, exceeding the recommended threshold of 0.60. The average variance extracted (AVE) values range from 0.884 to 0.913, surpassing the minimum criterion of 0.50. These results indicate that the convergence validity is satisfactory [58,59], the results showed that the reliability and the convergent validity was sufficient (Table 2). The results of the path analysis indicate a statistically significant relationship between the latent variables. The discriminative validity table (Table 3) demonstrates that the square root of the average variance extracted (AVE) values of the latent variables exceed the correlation coefficients among the different latent variables. This indicates that the discriminant validity is valid and aligns with the established reference standard [58]. Overall, the measuring model exhibited satisfactory levels of reliability, convergent validity, and discriminant validity. Furthermore, the obtained data was found to be suitable for application within the measurement model.

**Table 2:** Results of the confirmative factor analysis.

Constructs and scale items	Factor loading	CR	AVE
A1 I really enjoyed my time at the tea culture homestay	0.788	0.884	0.603
A2 Staying at a tea culture homestay is a meaningful activity for me	0.737		
A3 Staying at a tea culture homestay is very importance to me	0.827		
A4 I particularly like staying at the tea culture homestay	0.799		
A5 Staying at a tea culture homestay is closely related to me	0.728		
B1 I have a strong sense of belonging to a tea culture homestay	0.665	0.900	0.563
B2 I have a good impression of tea culture homestay	0.764		
B3 I feel a sense of belonging to tea culture	0.769		
B4 When I stay at a tea culture homestay, I can feel the cultural connotations displayed by the homestay staff	0.781		
B5 I like the traditional culture of the tea culture homestay	0.772		
B6 I want to know about the representative traditional tea culture homestay	0.722		
B7 I love the cultural atmosphere of the tea culture homestay	0.771		
C1 I intend to staying at a tea culture homestay	0.856	0.913	0.724
C2 I’m interested in staying at a tea culture homestay	0.851		
C3 I will recommend and choose the tea culture homestay	0.849		
C4 I have a high probability of staying at a tea culture homestay in the future	0.847		

**Table 3:** Test of discriminant validity of the constructs.

	Consumer involvement	Cultural identity	Purchase intention
Consumer involvement	0.603***		
Cultural identity	0.696***	0.563***	
Purchase intention	0.592***	0.722***	0.724***
AVE square root	0.777	0.750	0.851

Note: The numbers in the diagonal row (bold) are the average variance extracted by each latent construct. The numbers above diagonal are the squared correlation coefficients between the constructs.***p < 0.001.

### 4.3. Correlation analysis

Using AMOS 24.0 to carry out hypothesis testing on the research model, the results show that: the path coefficients of consumer involvement on the two variables of consumer purchase intention and cultural identity are 0.173 and 0.696, with p-values of 0.018 and 0.000, respectively, which proves that hypotheses H1 and H2 are valid, and that consumer involvement has the most obvious effect on cultural identity; there is a mediating relationship between consumer involvement and purchase intention there is a mediating relationship between the existence of cultural identity. In the case that the path coefficient of consumers’ willingness to purchase is 0.602, p ≤ 0.001, cultural identity has a significant positive influence on it, which further verifies the correctness of hypothesis H3.

### 4.4. Tests of mediating effects of cultural identity

Based on the judgment criteria and testing procedures of the mediating effect, the test results of the mediating effect of cultural identity are shown in the table, and the prediction of consumers’ purchase intention was found to be significant (t = 10.500, p < 0.001) after taking into account the influence of the mediating variable of cultural identity (t = 9.407, p < 0.001). Consumers’ involvement was significant in predicting cultural identity (t = 13.999, p < 0.001), and at the same time, cultural identity was significant in positively predicting consumers’ purchase intention (t = 3.351, p < 0.001). The above results indicate the mediating role of cultural identity between consumer involvement and consumer purchase intention(Table 4).

**Table 4:** Tests of Mediating Effects of Cultural Identity.

Regression equation		Fitness index			Significance	
Outcome variable	predictor variable	R	R^2^	F	t	p
Purchase intention	consumer involvement	0.520	0.270	110.259	10.500	0.000
Cultural identity	consumer involvement	0.630	0.397	195.961	13.999	0.000
Purchase intention	cultural identity	0.662	0.438	115.560	3.351	0.001
	consumer involvement				9.407	0.000

In addition to this, consumer purchase intention is not only directly affected by their involvement, but also by the mediating effect of cultural identity, so the upper and lower bounds of the bootstrap 95% confidence interval do not contain zero, which indicates that consumer involvement not only directly predicts consumer purchase intention, but also serves as an effective mediating factor, and can also be predicted by the mediating effect of cultural identity to consumer purchase intention, that is, there is a partial mediating effect of cultural identity between consumer involvement and consumer purchase intention, which proves the hypothesis H4(Table 5).

**Table 5:** Breakdown of Total, Direct, and Mediating Effects.

	Efficiency value	Boot standard error	BootCI lower limit	BootCI Upper Limit	Relative effect value
Aggregate effect	0.584	0.056	0.475	0.694	100%
Direct effect	0.211	0.063	0.087	0.335	36%
Mediating effect	0.373	0.063	0.248	0.500	64%

## 5. Conclusions and discussion

### 5.1. Conclusions

Based on the review and integration of a large number of related literates, this paper, based on the involvement theory and cultural identity theory, thoroughly explores the interaction between involvement and purchase intention from the aspects of cognitive involvement and emotional involvement, and introduces the three dimensions of cultural identity as the mediating variables, constructing a structural equation model for describing the complex relationship between involvement, cultural identity and purchase intention. By analyzing the recovered questionnaire data, we verified the constructed model and related hypotheses, and drew the following conclusions.After the analysis and model verification of this research, H1-H4 have been verified.

### 5.2. Discussion

Generation Z Involvement Can Positively Influence Generation Z Consumers’ Purchase Intention of Tea Culture homestay. The empirical test found that the two aspects of involvement studied in this paper: cognitive involvement and affective involvement have a positive predictive effect on generation Z’s purchase intention of tea culture homestay. With the gradual deepening of generation Z consumers’ knowledge of tea culture homestay, they will pay more attention to the collection and understanding of homestay information, and thus are more inclined to choose homestay, because they believe that understanding homestay can help to realize their goals; the emotion of generation Z affects the tendency to satisfaction and willingness to buy, and with a certain amount of emotion, generation Z of consumers’ emotion for homestay arises, so that generation Z of consumers will experience a pleasurable experience, which will prompt generation Z consumers’ purchase intention and emotional dependence on the homestay. Generation Z consumers with higher involvement degree will show stronger willingness to buy homestay. The lower involvement level will make generation Z consumers pay less attention to the information of homestay.

Generation Z Involvement Can Positively Influence Generation Z Consumers’ Cultural Identity of Tea Culture homestay. The empirical test found that the degree of involvement studied in this paper has a positive predictive effect on cultural identity. A higher degree of involvement among generation Z consumers will produce a strong sense of cultural identity for tea culture homestay. Generation Z consumers have a certain degree of involvement in the homestay, and after a certain degree of cognitive involvement and emotional involvement, their sense of identification with the homestay will be strengthened, and generation Z consumers will have a certain degree of cultural identification with the homestay as they continue to be involved in the homestay. A lower degree of involvement will make generation Z pay less attention to the cultural connotation of the homestay and fail to produce a sense of cultural identity to the homestay.

Generation Z consumers’ cultural identification with tea culture homestay can positively influence consumers’ purchase intention. Through empirical tests, it is found that the cultural identity studied in this paper has a positive predictive effect on consumers’ purchase intention. When generation Z consumers know more and more about the homestay and develop a love for it, and want to put it into practice, generation Z consumers are more likely to become sticky after living among the homestay and enjoying the life in the homestay, and will have a willingness to buy the homestay or the same type of living environment.

Cultural Identity as a Mediator in Generation Z Consumer Involvement and Consumer Purchase Intention. Among the previous studies, less content will take cultural identity as a mediating variable. Under the influence of a certain degree of involvement, generation Z of consumers will have a certain cultural identity towards homestay, which in turn will cause generation Z of consumers to purchase. Generation Z consumers believe that they have a certain cultural identity, and are more likely to generate consumer purchase intention after cognitive involvement and emotional involvement in homestay. The stronger the sense of cultural identity of generation Z consumers, the stronger their willingness to buy homestay. The deeper the involvement of generation Z consumers in homestay, the stronger the cultural identity of generation Z will be, which will increase the purchase intention of generation Z consumers.

### 5.3. Implications

Homestay is a form of cultural expression, the degree of involvement of generation Z consumers will affect the consumers’ willingness to buy, the cultural identity of the homestay affects the consumers’ willingness to buy, and the cultural embodiment of the homestay is also used as one of the important criteria for generation Z consumers to choose the homestay. From the conclusion of the study, this paper discusses the measures that homestay can take to maximize the possibility of stimulating the purchase intention of generation Z consumers.

Increase publicity and carry out activities with local culture to strengthen the cognition of generation Z consumers. It is possible to publicize the homestay through multiple channels to increase the visibility of the homestay, such as Xiaohongshu, Jieyin, etc., and use these tools to disseminate information related to the homestay and increase the involvement of generation Z of consumers. At the same time to carry out some activities related to local culture, through the way of the Internet to spread, in the Internet communication, the dissemination of the surface will be promoted, so that more people pay attention to the activities held in the homestay, pay attention to the cultural heritage contained in the homestay, and at the same time to attract people who are interested in the local culture to participate in the activities of the homestay. For example, the tea culture homestay can be in the small red book and other network platforms, to promote their own homestay, and can organize tea tasting, tea picking, tea production and other tea garden experience projects and other activities, and publicity in the network, to attract consumer interest in the homestay. Let generation Z of consumers in the network on the homestay to produce a certain degree of cognitive involvement and emotional involvement, to attract generation Z of consumers to produce the willingness to buy.

Open themed special rooms to create homestay products with local cultural characteristics. When the homestay room is arranged, it can be combined with local cultural characteristics to decorate the homestay, so that the appearance of the homestay has a certain cultural connotation. In the selection of homestay products, adjustments can be made according to local characteristics. For example, in the tea culture homestay set up some teamaking tools, and baked tea in the room, so that the room is full of tea aroma, in the homestay products can be introduced into the tea elements, tea culture is embedded in the overall decoration of the homestay. homestay is a reflection of the local culture, to create a theme room, the use of local characteristics in the room products will be more attractive to generation Z of consumers.

Enhance the cultural awareness of service staff and open chat rooms for hosts and guests. Simply from the appearance of the homestay to reflect the local culture is often not enough, culture is more focused on the subtle influence, and for the direct face to face guests of the service staff, their cultural awareness is also very important. When the service personnel can reflect the local folklore in the process of conversation with guests, it is also very important for guests to understand the local cultural characteristics. The opening of the host-guest chat room can allow guests to travel in the leisure time, sit down and slowly feel the local culture, the host of the homestay can be face-to-face with the guests to communicate, you can learn about the local cultural characteristics, the origins of the homestay, and so on, the existence of the chat room provides a place for communication between the host and the guest. For example, in Wuyishan homestay, the store manager will talk to the guests about the origin of the homestay, homestay tea space atmosphere to create a good environment, the layout of the exquisite, clean, full of interest, rich in cultural sense, there are specialized tea masters tea, tea culture elements into the process of service, while the hosts and guests can be in the homestay tea space on the homestay, the local culture to understand and feel.

Launch of homestay peripherals related to local culture and cooperation with popular local drink stores. In the past, tourists would buy souvenirs from local souvenir stores. Setting up a boutique house inside the homestay and arranging some peripheral products that combine local culture with the homestay, such as blind boxes, jewelry and other products, these knickknacks can be easily carried around and presented to the guests at an affordable price. The purchase of the products by the residents allows the culture to enter their lives, and with the logo of the homestay on these peripherals, a certain amount of visibility will be brought about when the residents carry these products with them. Putting pamphlets about the homestay in popular local beverage stores, where guests can browse the pamphlets while waiting for their drinks, and the pamphlets contain information about famous attractions, travel routes, and homestay, will increase the rate of staying at the homestay.

### 5.4. Limitations and future research

In terms of data collection, this paper mainly distributes questionnaires online, and there is insufficient coverage of data samples, because the sample capacity is only 300 and cannot cover various age levels, education levels and other attributes, and the conclusions of the study may not be persuasive. Therefore, the selection of the sample may lack a certain degree of relevance and typicality. In the future research, scholars can expand the sample capacity and extend the scope of the sample in terms of age level, education level and income attributes, so that the overall sample of the study can be more representative.

The research in this paper mainly adopts quantitative analysis methods, but the degree of consumer involvement, cultural identity and consumer purchase intention are to a certain extent individual subjective. The use of quantitative analysis can only be analyzed from a certain aspect and lacks generality. In the future, scholars can use a combination of analytical methods to make the findings more generalized.

This paper focuses on the purchase intention of generation Z of consumers, but the situation in different fields is different and cannot be generalized, therefore, the conclusions drawn in this paper and whether the management insights are applicable to other fields need to be studied in depth.
